# FCERI and Histamine Metabolism Gene Variability in Selective Responders to NSAIDS

**DOI:** 10.3389/fphar.2016.00353

**Published:** 2016-09-29

**Authors:** Gemma Amo, José A. Cornejo-García, Jesus M. García-Menaya, Concepcion Cordobes, M. J. Torres, Gara Esguevillas, Cristobalina Mayorga, Carmen Martinez, Natalia Blanca-Lopez, Gabriela Canto, Alfonso Ramos, Miguel Blanca, José A. G. Agúndez, Elena García-Martín

**Affiliations:** ^1^Departamento de Farmacología, Universidad de ExtremaduraCáceres, Spain; ^2^Laboratorio de Investigación, Instituto de Investigación Biomédica de Málaga, Hospital Regional Universitario de Málaga, Universidad de MálagaMálaga, Spain; ^3^Servicio de Alergologia, Hospital Infanta CristinaBadajoz, Spain; ^4^UGC de Alergia, Instituto de Investigación Biomédica de Málaga, Hospital Regional Universitario de Málaga, Universidad de MálagaMálaga, Spain; ^5^Servicio de Alergologia, Hospital Infanta LeonorMadrid, Spain; ^6^Departamento de Matemáticas, Universidad de ExtremaduraCáceres, Spain

**Keywords:** Fcε RI, histamine, non-steroidal anti-inflammatory drugs (NSAIDS), hypersensitivity drug reactions, biomarkers

## Abstract

The high-affinity IgE receptor (Fcε RI) is a heterotetramer of three subunits: Fcε RIα, Fcε RIβ, and Fcε RIγ (α*βγ*2) encoded by three genes designated as *FCER1A, FCER1B* (*MS4A2*), and *FCER1G*, respectively. Recent evidence points to FCERI gene variability as a relevant factor in the risk of developing allergic diseases. Because Fcε RI plays a key role in the events downstream of the triggering factors in immunological response, we hypothesized that FCERI gene variants might be related with the risk of, or with the clinical response to, selective (IgE mediated) non-steroidal anti-inflammatory (NSAID) hypersensitivity. From a cohort of 314 patients suffering from selective hypersensitivity to metamizole, ibuprofen, diclofenac, paracetamol, acetylsalicylic acid (ASA), propifenazone, naproxen, ketoprofen, dexketoprofen, etofenamate, aceclofenac, etoricoxib, dexibuprofen, indomethacin, oxyphenylbutazone, or piroxicam, and 585 unrelated healthy controls that tolerated these NSAIDs, we analyzed the putative effects of the FCERI SNPs *FCER1A* rs2494262, rs2427837, and rs2251746; *FCER1B* rs1441586, rs569108, and rs512555; *FCER1G* rs11587213, rs2070901, and rs11421. Furthermore, in order to identify additional genetic markers which might be associated with the risk of developing selective NSAID hypersensitivity, or which may modify the putative association of FCERI gene variations with risk, we analyzed polymorphisms known to affect histamine synthesis or metabolism, such as rs17740607, rs2073440, rs1801105, rs2052129, rs10156191, rs1049742, and rs1049793 in the *HDC, HNMT*, and *DAO* genes. No major genetic associations with risk or with clinical presentation, and no gene-gene interactions, or gene-phenotype interactions (including age, gender, IgE concentration, antecedents of atopy, culprit drug, or clinical presentation) were identified in patients. However, logistic regression analyses indicated that the presence of antecedents of atopy and the DAO SNP rs2052129 (GG) were strongly related (*P* < 0.001 and *P* = 0.005, respectively) with selective hypersensitivity to ibuprofen. With regard to patients with selective hypersensitivity to ASA, men were more prone to develop such a reaction than women (*P* = 0.011), and the detrimental *DAO* SNP rs10156191 in homozygosity increased the risk of developing such hypersensitivity (*P* = 0.039).

## Introduction

Type B drug-induced hypersensitivity reactions (DHR) occur only in susceptible individuals with a frequency of 5–10% of all adverse drug reactions (Khan and Solensky, 2010). In general, these reactions are severe and occasionally may be life-threatening. In recent years evidence has accumulated to support the notion that DHR may be caused by various mechanisms and is the result of a complex multifactorial and multigenic process (Pirmohamed, 2006). Regarding culprit drugs, non-steroidal anti-inflammatory drugs (NSAIDs) are among the most frequent causes of DHR together with antibiotics (Cornejo-Garcia et al., 2009; Doña et al., 2011, 2012; Kowalski et al., 2011). Hypersensitivity reactions to a single NSAID (selective reactions) are the result of an immunological mechanism, which is either IgE-mediated in acute reactions or T cell-mediated in delayed reactions. Clinical manifestations include cutaneous reactions, respiratory reactions and anaphylaxis, which may appear with different onset times after drug intake. Histamine release from mast cells after IgE receptor activation plays a relevant role in allergic inflammation and in the development of clinical symptoms (Mita et al., 2001; Kowalski et al., 2011).

*A priori*, genetic variations affecting both components (expression and/or function) of this signaling pathway, including high affinity IgE receptor and histamine metabolizing enzymes, could modify the risk of developing hypersensitivity to NSAIDs, and/or the presentation of clinical manifestations. In fact, recent studies have demonstrated an association between several allergic diseases including drug hypersensitivity and polymorphisms in these genes (García-Martín et al., 2006, 2007a; Kennedy et al., 2008; Gervasini et al., 2010; Maintz et al., 2011).

The high-affinity IgE receptor (Fcε RI) is a heterotetramer of three subunits: Fcε RIα, Fcε RIβ, and Fcε RIγ (α*βγ*_2_) encoded by three genes designated as *FCER1A, FCER1B* (*MS4A2*), and *FCER1G*, respectively. The genes coding for all FcεRI subunits are polymorphic and some of these polymorphisms have been associated with several atopic diseases (MacGlashan et al., 1998, 1999; Saini et al., 1999; Weidinger et al., 2008; Zhang et al., 2010; Li et al., 2014). The *FCER1A* gene is located on chromosome 1q23 (1:159259504-159278014; 1000 Genomes, 2015), and it encodes a protein with two extracellular IgE-like domains with ligand-binding sites (Potaczek and Kabesch, 2012). The *FCER1B* gene is located on chromosome 11q12-13 (11:59855734–59863444; 1000 Genomes, 2015), and it encodes a 244-amino acid protein with a non-canonic intracellular ITAM domain. Functionally, it is a signal-augmenting subunit. The *FCER1G* gene is located on chromosome 1q23 (1:161185024–161190489; 1000 Genomes, 2015) and encodes an 86-amino acid protein. Functionally, it is a signal-transducing subunit and plays an essential role in the induction of mast cell degranulation and survival (Manikandan et al., 2012).

With regard to histamine homeostasis, histamine biosynthesis is catalyzed by the enzyme L-histidine decarboxylase (HDC, E.C. 4.1.1.22). Two enzymes participate in the degradation of histamine: Histamine N-methyltransferase (HNMT, E.C. 2.1.1.8) and diamine oxidase (DAO; E.C. 1.4.3.6; García-Martín et al., 2009). There is high individual variability in histamine metabolism which is, at least in part, genetically determined, although other factors, such as gender (García-Martín et al., 2007b) play a major role in the variability of histamine metabolism. An association between polymorphisms of histamine metabolizing enzymes and the clinical presentation of allergic diseases has been demonstrated (García-Martín et al., 2006, 2007a; Kennedy et al., 2008; Gervasini et al., 2010; Maintz et al., 2011).

The *HDC* gene is located in chromosome 15q21-q22 and spans around 24 kilobases. The *HNMT* gene is located in chromosome 2q22.1. The human *DAO* gene spans ~10 kb and is located in chromosome 7q34-q36. A promoter SNP and three common non-synonymous SNPs have been identified in Caucasian individuals, and the functional effects of these SNPs in enzyme activity have been studied in detail (Ayuso et al., 2007; García-Martín et al., 2007b).

In order to investigate the role of genetic factors in the risk of developing selective NSAID hypersensitivity, both isolated and combined, given that the clinical outcome depends on the interplay of IgE response and the consequent release of mediators, in this study we analyzed functional polymorphisms in high affinity IgE receptors and genes related to histamine metabolism in a large group of well-phenotyped patients suffering from single-NSAID-induced hypersensitivity reactions. Our aim was to elucidate the putative association of these gene polymorphisms with several clinical phenotype parameters, including gender, age, antecedents of atopy, culprit drug, IgE levels and clinical presentation, and to analyze putative gene-gene interactions. The final goal was to identify biomarkers which alone or combined with pharmacogenomics or metabolic biomarkers (Agúndez et al., 2009a,b, 2015; Cornejo-García et al., 2012; Martinez et al., 2014; García-Martín et al., 2015a; Ariza et al., 2016) might be useful in prevention, diagnosis and/or proper management of these patients.

## Patients and methods

### Study population

We studied a cohort of 899 individuals, consisting of 314 unrelated Spanish patients with single-NSAID-induced hypersensitivity reactions and 585 unrelated healthy controls. Written consent for participation was obtained for all participants. All the patients who were invited to participate in the study agreed to do so. Of the patients, 145 were recruited from the Allergy service, Hospital Infanta Leonor (Madrid, Spain), 100 were recruited from the Allergy service, Regional Hospital (Málaga, Spain) and 69 were recruited from the Allergy Department, Infanta Cristina Hospital (Badajoz, Spain). Diagnosis was carried out as previously described (Doña et al., 2011). After confirming good tolerance to a full therapeutic dose of ASA, incremental doses of the culprit drug were given until the therapeutic dose for the analgesic/NSAIDs implicated in the study was achieved. In the case of selective responses to ASA, confirmation of the selective response was made by challenge with indomethacin as reported elsewhere (Doña et al., 2011).

Participants' characteristics are summarized in Table 1. The NSAIDs responsible for the reaction are summarized in Table 2. Clinical presentation distributed according to gender and culprit drug are shown in Table 3.

**Table 1:** **Characteristics of the study group**.

	**Patients (*n* = 314)**	**Healthy subjects (*n* = 585)**
Women, *n* (%)	205 (65.3)	356 (60.8)
Age [*SD*; range]	45.4 [16.1; 5–82]	22.3 [4.8; 20–57]
Antecedents of atopy, *n* (%)	66 (21.0)	0
Antecedents of urticaria, *n* (%)	4 (1.3)	0
Interval <1 h; *n* (%), Single NSAID-induced urticaria/angioedema, and anaphylaxis	232 (73.9)	n.a.
Interval >24 h; *n* (%), Single NSAID-induced delayed reaction	13 (4.1)	n.a.
Interval unknown, *n* (%)	69 (22.0)	n.a.

**Table 2:** **Culprit drug for selective NSAID-induced hypersensitivity**.

**Culprit drug**	**Total No. (%)**	**PATIENTS DISTRIBUTED BY GENDER**	
		**Men (No. %)**	**Women (No. %)**
Metamizole	108 (34.4)	32 (29.3)	76 (37.1)
Ibuprofen	94 (29.9)	35 (32.1)	59 (28.8)
Diclofenac	35 (11.1)	15 (13.8)	20 (9.8)
Paracetamol	20 (6.4)	7 (6.4)	13 (6.3)
Acetyl salicylic acid	19 (6.1)	8 (7.3)	11 (5.4)
Propifenazone	12 (3.8)	4 (3.7)	8 (3.9)
Naproxen	9 (2.9)	3 (2.8)	6 (2.9)
Ketoprofen	3 (1.0)	0	3 (1.5)
Dexketoprofen	3 (1.0)	2 (1.8)	1 (0.5)
Etofenamate	3 (1.0)	1 (0.9)	2 (1.0)
Aceclofenac	1 (0.3)	1 (0.9)	0
Etoricoxib	1 (0.3)	0	1 (0.5)
Dexibuprofen	1 (0.3)	0	1 (0.5)
Indomethacin	1 (0.3)	0	1 (0.5)
Oxyphenbutazone	1 (0.3)	0	1 (0.5)
Piroxicam	1 (0.3)	0	1 (0.5)
Unknown	2 (0.6)	1 (0.9)	1 (0.5)
Total	314 (100)	109 (100)	205 (100)

**Table 3:** **Clinical presentation of selective NSAID-induced hypersensitivity**.

**Gender**	**Urticaria + Angioedema (No. %)**	**Anaphylaxis (No. %)**	**Exanthema (No. %)**	**Mixed pattern (No. %)**	**Respiratory (No. %)**	**Toxic hepatitis (No. %)**	**Unknown (No. %)**	**Total (No. %)**
**PATIENTS DISTRIBUTED BY CLINICAL PRESENTATION**								
Men	57 (52.3)	40 (36.7)	9 (8.3)	2 (1.8)	0	0	1 (0.9)	109 (100)
Women	113 (55.1)	68 (33.2)	10 (4.9)	7 (3.4)	5 (2.4)	1 (0.5)	1 (0.5)	205 (100)
**Culprit drug**	**Urticaria** + **Angioedema (No. %)**	**Anaphylaxis (No. %)**	**Exanthema (No. %)**	**Mixed pattern (No. %)**	**Respiratory (No. %)**	**Toxic hepatitis (No. %)**	**Unknown (No. %)**	**Total (No. %)**
Metamizole	45 (41.7)	53 (49.1)	7 (6.5)	3 (2.8)	0	0	0	108 (100)
Ibuprofen	70 (74.5)	18 (19.1)	2 (2.1)	3 (3.2)	1 (1.1)	0	0	94 (100)
Diclofenac	13 (37.1)	20 (57.1)	2 (5.7)	0	0	0	0	35 (100)
Paracetamol	12 (60.0)	5 (25.0)	1 (5.0)	0	2 (10.0)	0	0	20 (100)
Acetyl Salicylic Acid	13 (68.4)	1 (5.3)	2 (10.5)	0	2 (10.5)	0	1 (5.3)	19 (100)
Propifenazone	6 (50.0)	4 (33.3)	1 (8.3)	1 (8.3)	0	0	0	12 (100)
Naproxen	4 (44.4)	2 (22.2)	1 (11.1)	2 (22.2)	0	0	0	9 (100)
Ketoprofen	2 (66.7)	0	0	0	0	1 (33.3)	0	3 (100)
Dexketoprofen	1 (33.3)	2 (66.6)	0	0	0	0	0	3 (100)
Etofenamate	2 (66.7)	0	1 (33.3)	0	0	0	0	3 (100)
Aceclofenac	0	1 (100)	0	0	0	0	0	1 (100)
Etoricoxib	1 (100)	0	0	0	0	0	0	1 (100)
Dexibuprofen	0	1 (100)	0	0	0	0	0	1 (100)
Indomethacin	0	0	1 (100)	0	0	0	0	1 (100)
Oxyphenbutazone	0	0	0	0	0	0	1 (100)	1 (100)
Piroxicam	1 (100)	0	0	0	0	0	0	1 (100)
Unknown	0	1 (50)	1 (50)	0	0	0	0	2 (100)
Total	170	108	19	9	5	1	2	314

The healthy controls were recruited from staff and medical students of the Hospitals and the Universities participating in the study, and were ethnically matched with patients (all were unrelated Spanish individuals). All control subjects tolerated the NSAIDs most frequently involved in selective hypersensitivity, as shown in Table 2. Specifically, all control individuals previously received metamizole, ibuprofen, diclofenac, paracetamol, and ASA, and experienced no hypersensitivity or other ADRs. Individuals with the above-mentioned characteristics were asked to participate, and 97% of them agreed. A medical history was obtained and an examination was performed for each participant to exclude pre-existing disorders. Individuals with familial (up to second-degree relatives) or personal antecedents of allergic, atopic, or autoimmune diseases were excluded from the control group to avoid confounders. The protocol for this study was in accordance with the Declaration of Helsinki and its subsequent revisions and was approved by the respective Ethics Committees of the participating Hospitals.

### Genotype analysis

Genomic DNA was obtained from peripheral leukocytes and purified in accordance with standard procedures. The SNPs analyzed were selected according to allele frequencies (over 0.01) in the study population, and either functional or clinical relevance, in line with published evidence (Preuss et al., 1998; García-Martín et al., 2006, 2007a, 2009; Ayuso et al., 2007; Maintz et al., 2011; Amo et al., 2016), and the public 1000 genomes database release of 17 Nov. 2015. Genotyping was performed by TaqMan assays (Life Technologies, Alcobendas, Madrid, Spain). Details of the TaqMan probes and the allele frequencies in Caucasian individuals are summarized in Table 4. We studied nine *FCER1* SNPs, as well as SNPs corresponding to genes involved in histamine synthesis (*HDC*) or metabolism *(HNMT and DAO*). All these histamine-related SNPs have demonstrated functional and/or clinical implications (García-Martín et al., 2006, 2007a,b, 2009, 2015b; Ayuso et al., 2007; Gervasini et al., 2010; Agúndez et al., 2012).

**Table 4:** **SNPs analyzed in this study**.

**Gene**	**Chromosomal location**	**dbSNP**	**Consequence**	**Assay ID**	**MAF (1000 genomes, European individuals)**	Statistical power (two tailed, OR = 1.5, α = 0.05)^*^ (%)
*FCER1A*	1:159253672	rs2494262 A/C	Upstream gene	C____494924_20	0.44 C	98
*FCER1A*	1:159258545	rs2427837 G/A	Upstream gene	C__16233438_20	0.30 A	96
*FCER1A*	1:159272060	rs2251746 T/C	Intronic	Custom-designed	0.30 C	96
*FCER1B*	11:59856028	rs1441586 T/C	5 prime UTR	C___1842226_10	0.46 C	98
*FCER1B*	11:59863104	rs569108 A/G	Missense 237 E/G	C____900116_10	0.04 G	36 (a)
*FCER1B*	11:59863253	rs512555 C/T	3 prime UTR	C___7513065_10	0.04 T	37 (b)
*FCER1G*	1:161184875	rs11587213 A/G	Upstream gene	C__27848237_10	0.18 G	88
*FCER1G*	1:161185058	rs2070901 G/T	Non-coding transcript exon	C__15867981_20	0.27 T	97
*FCER1G*	1:161188936	rs11421 T/C	3 prime UTR	C___1841966_1_	0.15 C	91
*HNMT*	2:138759649	rs11558538 C/T	Missense 105 T/I	C__11650812_20	0.10 T	73 (c)
*DAO*	7:150548972	rs2052129 G/T	Upstream gene	C__11630976_1	0.25 T	97
*DAO*	7:150553605	rs10156191 C/T	Missense 16 T/M	C__25593951_10	0.27 T	96
*DAO*	7:150554553	rs1049742 C/T	Missense 332 S/F	C___7599782_20	0.08 T	58 (d)
*DAO*	7:150557665	rs1049793 C/G	Missense 645 H/D	C___7599774_10	0.27 G	97
*HDC*	15:50534514	rs2073440 T/G	Missense 644 E/D	C__15950871_20	0.02 G	31 (e)
*HDC*	15:50555544	rs17740607 G/A	Missense 31 T/M	C__25624415_20	0.10 A	69 (f)

* *The statistical power (two tailed, OR = 2.0, α = 0.05) is as follows: (a) 82%; (b) 83%; (c) 100%; (d) 97%; (e) 75%; (f) 99%. MAF, Minor allele frequency*.

Detection was carried out by means of real-time PCR (qPCR) in an Eppendorf realplex thermocycler using fluorescent probes. The amplification conditions were as follows: After a denaturation time of 10 min at 96°C, 45 cycles of 92°C 15 s 60°C 90 s were carried out and fluorescence was measured at the end of each cycle and at endpoint. All samples were determined in triplicate and genotypes were assigned both by means of gene identification software (RealPlex 2.0, Eppendorf), and by analysis of the reference cycle number for each fluorescence curve, calculated using the CalQPlex algorithm (Eppendorf).

### Statistical analyses

Statistical power for each SNP was evaluated with a genetic model to analyze the frequency for the minor allele with an odds ratio (OR) value = 1.5 (α = 0.05) based on the allele frequencies observed in the control group. Table 4 shows the statistical power for each SNP analyzed. For most SNPs the statistical power was very high. In some cases, because of the low minor allele frequency observed, the statistical power was not sufficient to detect an OR = 1.5 but was sufficient to detect an OR = 2.0 with a bilateral power of more than 80% (Table 4), with the single exception of the HDC SNP rs2073440 T/G, whose power was sufficient to detect an OR = 2.1.

SNPStats software (Solé et al., 2006) was used to calculate allele and genotype frequencies, to analyze the Hardy-Weinberg equilibrium, and to determine linkage disequilibrium statistics and haplotype frequency estimation. Multiple comparison adjustment was done by using the False Discover Rate (FDR) correction (http://www.sdmproject.com/utilities/?show=FDR). Analyses of association with a response variable (culprit drugs and reactions) based on logistic regression were performed using SPSS 21.0 for Windows. For these analyses, we also determined the odds ratio and the corresponding 95% confidence intervals. The Hosmer-Lemeshow goodness of fit test for logistic regression was used. For the IgE response variable a multiple linear regression model was calculated. The results were considered as statistically significant when the *p*-value was less than 0.05.

## Results

The percentage of women was slightly higher in the cases, as compared to the control individuals (Table 1), although no statistically significant gender differences between cases and controls were present (Chi-square *P* = 0.191). Conversely, age was lower in the controls than in the cases. However, age is not a key factor in this study, as all the control individuals were tolerant to NSAIDs and the odds are extremely low that they may eventually develop selective NSAID-induced hypersensitivity.

The most common culprit drugs for selective NSAID-induced hypersensitivity were metamizole, ibuprofen, diclofenac, paracetamol, and ASA (Table 2). Although the frequency for metamizole-induced hypersensitivity was higher in women than in men, OR = 1.42 and 95% confidence interval (CI) 0.84–2.41; *P* = 0.171, the frequency difference was not statistically significant, and neither were the differences for gender-related frequencies for the rest of the NSAIDs included in Table 2. These frequencies correspond to those previously described by our group (Doña et al., 2011; Blanca-López et al., 2016a,b).

The most frequent clinical presentation was urticaria + angioedema, followed by anaphylaxis, exanthema and mixed pattern. No gender-related differences in clinical presentation were observed (Table 3). Clinical presentation, however, was strongly related with the culprit drug: The clinical presentation urticaria + angioedema was particularly frequent when the culprit drug was ibuprofen (OR = 3.50, 95% CI = 1.99–6.19); *P* <0.001. In contrast, when the causative drug was metamizole or diclofenac, the most common clinical presentation was anaphylaxis (OR = 2.65, 95% CI = 1.58–4.44; *P* < 0.001) and (OR = 2.89, 95% CI = 1.34–6.28, *P* = 0.003), respectively. When the culprit drug was paracetamol or ASA the most frequent presentation was urticaria + angioedema, although for these two drugs the association of the drug with clinical presentation was not statistically significant. These phenotypic features correspond to those previously reported for selective NSAID hypersensitivity patients (Cornejo-Garcia et al., 2009; Doña et al., 2011; García-Martín et al., 2015a).

*FCER1* genotyping results are summarized in Table 5. We checked the codominant, dominant, recessive, overdominant and additive models and the best fit was obtained with the recessive model. *FCER1* SNPs did not show statistically significant differences when patients and controls were compared in any of the genetic models analyzed. The genotyping results related to histamine synthesis and metabolism genes are summarized in Table 6. Once again, the best fit for histamine metabolism genes was obtained with the recessive model. Statistically significant differences on comparing cases and control subjects were identified for the *DAO* SNP rs10156191, which caused decreased enzyme activity (Ayuso et al., 2007). The observed difference was related to the frequency of homozygous individuals for the minor allele, which obtained a marginal significance that was not observed when the allele frequency (instead of the genotypes) was analyzed. When correction for multiple comparisons was carried out by using FDR, the P value for the recessive model for the DAO rs10156191 SNP was not significant (corrected *P* = 0.294), whereas the *P*-value for carriers of the minor allele for the HDC SNP rs2073440 remained significant (corrected *P*-value = 0.021).

**Table 5:** *****FCER1*** SNPs analyzed in this study**.

**Gene**	**Chromosomal location**	**dbSNP**	**Cases non-mutated/heterozygous/homozygous**	**Cases MAF**	**Control non-mutated/heterozygous/homozygous**	**Control MAF**	**Comparison values (recessive model; OR, 95% CI)**	**Comparison values (carrier of the minor allele; OR, 95% CI)**
*FCER1A*	1:159253672	rs2494262	83/146/73	0.483	136/260/107	0.471	1.12 (0.75–1.67); *P* = 0.589	1.05 (0.86–1.29); *P* = 0.633
*FCER1A*	1:159258545	rs2427837	181/101/17	0.226	297/212/38	0.263	0.73 (0.40–1.34); *P* = 0.312	0.82 (0.65–1.03); *P* = 0.089
*FCER1A*	1:159272060	rs2251746	185/107/14	0.221	300/221/34	0.260	1.22 (0.82–1.81); *P* = 0.337	1.10 (0.90–1.34); *P* = 0.350
*FCER1B*	11:59856028	rs1441586	82/148/71	0.482	160/275/114	0.458	1.22 (0.82–1.81); *P* = 0.337	1.10 (0.90–1.34); *P* = 0.350
*FCER1B*	11:59863104	rs569108	274/21/1	0.039	517/39/0	0.035	5.66 (0.23–139.3; *P* = 0.170	1.11 (0.66–1.88); *P* = 0.690
*FCER1B*	11:59863253	rs512555	290/22/1	0.038	530/39/0	0.034	5.48 (0.22–134.9); *P* = 0.177	1.12 (0.67–1.89); *P* = 0.660
*FCER1G*	1:161184875	rs11587213	219/79/15	0.174	408/150/16	0.159	1.75 (0.85–3.60); *P* = 0.126	1.12 (0.86–1.45); *P* = 0.400
*FCER1G*	1:161185058	rs2070901	164/124/25	0.278	285/231/52	0.294	0.84 (0.50–1.40); *P* = 0.493	0.92 (0.74–1.14); *P* = 0.453
*FCER1G*	1:161188936	rs11421	217/85/10	0.168	390/157/24	0.180	0.75 (0.35–1.60); *P* = 0.452	0.93 (0.71–1.20); *P* = 0.553

*Three hundred and fourteen cases and 585 control individuals were included in the study. The sum of genotypes do not correspond to all cases and controls because of DNA shortage*.

**Table 6:** **SNPs related with histamine synthesis and degradation analyzed in this study**.

**Gene**	**Chromosomal location**	**dbSNP**	**Cases non-mutated/heterozygous/homozygous**	**Cases MAF**	**Control non-mutated/heterozygous/homozygous**	**Control MAF**	**Comparison values (recessive model; OR, 95% CI)**	**Comparison values (carrier of the minor allele; OR, 95% CI)**
*HNMT*	2:138002079	rs11558538	260/51/2	0.088	466/95/7	0.096	0.51 (0.11–2.48); *P* = 0.398	0.91 (0.65–1.28); *P* = 0.580
*DAO*	7:150851884	rs2052129	173/118/14	0.239	322/204/30	0.237	0.87 (0.45–1.68); *P* = 0.676	1.02 (0.80–1.27); *P* = 0.928
*DAO*	7:150856517	rs10156191	178/122/13	0.236	304/214/43	0.267	0.52 (0.27–0.99); *P* = 0.042	0.85 (0.68–1.06); *P* = 0.160
*DAO*	7:150857465	rs1049742	277/34/1	0.058	483/68/2	0.065	0.87 (0.08–9.66); *P* = 0.911	0.88 (0.58–1.33); *P* = 0.540
*DAO*	7:150860577	rs1049793	160/123/28	0.288	292/220/51	0.290	1.00 (0.61–1.65); *P* = 0.994	1.01 (0.81–1.25); *P* = 0.940
*HDC*	15:50242317	rs2073440	289/16/1	0.029	354/25/0	0.033	3.67 (0.15–90.52); *P* = 0.269	0.44 (0.26–0.77); *P* = 0.003
*HDC*	15:50263347	rs17740607	255/53/3	0.095	346/68/8	0.100	0.51 (0.13–1.94); *P* = 0.313	0.95 (0.67–1.35); *P* = 0.770

*Three hundred and fourteen cases and 585 control individuals were included in the study. The sum of genotypes do not correspond to all cases and controls because of DNA shortage*.

Table 7 shows the statistically significant interaction of the genotypes studied and gender. The association of the *FCER1A* SNP rs2427837 with the risk of developing NSAID-induced hypersensitivity showed a positive interaction with gender, the association being stronger in women (Table 7). A statistically significant, genotype-gender interaction was observed for two other *FCER1* genotypes (rs2251746 in women and rs11587213 in men). When FDR correction for multiple comparisons (both genders and all SNPs) was made, the corrected *P*-values remained significant: *P* = 0.045 for the genetic associations with the SNPs rs2427837 and rs2251746 and *P* = 0.035 for the SNP rs11587213.

**Table 7:** **Statistically significant gender-related risk associations**.

**Gene**	**Chromosomal location**	**dbSNP**	**Gender**	**Cases MAF**	**Control MAF**	**Comparison values (recessive model; OR, 95% CI)**	**Comparison values (carrier of the minor allele; OR, 95% CI)**
*FCER1A*	1:159258545	rs2427837	Women	0.198	0.279	0.64 (0.28–1.46); *P* = 0.280	0.64 (0.46–0.89); *P* = 0.007
*FCER1A*	1:159272060	rs2251746	Women	0.196	0.271	0.68 (0.28–1.63); *P* = 0.380	0.65 (0.47–0.91); *P* = 0.010
*FCER1G*	1:161184875	rs11587213	Men	0.229	0.129	2.42 (0.74–7.93); *P* = 0.135	2.01 (1.26–3.19); *P* = 0.003

Logistic regression analyses were carried out with separate models for each clinical presentation, as shown in Table 3, by comparing between cases all genotypes, gender, age, IgE concentration, and antecedents of atopy. In addition, we analyzed putative associations with response, stratifying patients into two groups: Single NSAID-induced urticarial/angioedema or anaphylaxis (SNIUAA) and single NSAID-induced delayed reactions (SNIDR). No significant associations were identified. In addition, logistic regression analyses were carried out with separate models for each culprit drug (only drugs with 19 or more cases were included, as shown in Table 2), by comparing between cases all genotypes, gender, age, IgE concentration, and antecedents of atopy.

For ibuprofen, the Hosmer–Lemeshow (HL) goodness of fit test was equal to 0.80 and revealed that age [*P* = 0.032; OR (95% CI) = 0.91 (0.83–0.99)], the presence of atopy antecedents [*P* < 0.001; OR = 19.61 (4.13–90.90)] and the DAO SNP rs2052129 (GG); [*P* = 0.005; OR = 13.25 (2.14–81.84)] were related to the risk of developing hypersensitivity. With regard to metamizole, although linear regression analysis suggested association with the absence of atopy (*P* = 0.008; OR = 5.80, 95% CI = 1.58–21.28), the HL goodness of fit test was equal to 0.001, and therefore the significance of these findings is limited. For ASA, we identified significant associations with the SNPs rs10156191 (TT) [*P* = 0.035, OR = 44.59; 95% CI = 1.22–1630.56] and with gender (for men, *P* = 0.011, OR = 25.64; 95% CI = 2.13–333.33) with an HL goodness of fit test equal to 0.957. No significant associations were observed with paracetamol or diclofenac. IgE levels did not show any association with phenomic or genomic markers. We did not identify any additional significant associations, although we cannot rule out association with other culprit drugs because the subgroup sizes were not sufficiently large to reach statistical significance.

## Discussion

NSAID-induced hypersensitivity type B adverse reactions are mediated by immunological and non-immunological mechanisms. Two major clinical phenotypes have been described: Selective NSAID hypersensitivity, which is drug-specific and an IgE-mediated mechanism, and cross-intolerance in which chemically non-related NSAIDs induce the reaction (Kowalski et al., 2013). Because selective hypersensitivity is an IgE-mediated mechanism, we analyzed genetic variations at the high-affinity IgE receptor, which has been shown to be related with allergic disorders (MacGlashan et al., 1998, 1999; Saini et al., 1999; Weidinger et al., 2008; Zhang et al., 2010; Li et al., 2014; Amo et al., 2016). It is to be noted that, despite the large body of published evidence supporting association of *FCER1* SNPs with allergic diseases, this is the first study to analyze the putative role of *FCER1* SNPs in selective NSAID hypersensitivity. In addition, we previously identified genetic factors related to cross intolerance (Agúndez et al., 2012) and, of these, one non-synonymous *DAO* gene variation, designated as rs10156191, was overrepresented among cross-intolerant patients, thus providing the basis for a detailed study on the role of genetic variations in histamine metabolism in patients with selective hypersensitivity to NSAIDs.

*FCER1* genotypes have been linked to ASA-intolerant asthma. In a study carried out on 126 Korean patients with ASA-intolerant asthma, Palikhe and co-workers analyzed six *FCER1* SNPs, five of which were also analyzed in our study (Palikhe et al., 2008a,b). They identified a weak association of the *FCER1G* rs11587213 SNP with ASA-intolerant asthma, the patients showing increased frequency for the AA genotype. The same study reported an association of the two *FCER1A* SNPs rs2427827 and rs2251746 with specific IgE levels and an association of the *FCER1G* SNP 11587213 with both total IgE and specific IgE levels.

Our findings do not support an association of these *FCER1A* genotypes with the risk of developing selective hypersensitivity to ASA, or any other NSAID included in this study, and neither do they support an association with total IgE levels. Potential discrepancies between our study and that of Palikhe and co-workers may arise from the differences in clinical presentation, differences in the number of patients, and the different ethnic origin of patients and controls. In fact, in the study by Palikhe et al. (2008a,b) the allele frequencies observed in Korean individuals differ considerably from those reported in this study and in the 1000 genomes website for individuals of Caucasian descent (see Table 4).

Additional clinical associations for *FCER1A* SNPs are the putative association of the SNP rs2298804 with the risk of developing systemic lupus erythematosus in a study carried out in China (Yang et al., 2013), and the association of the SNP rs2298805 with the risk of developing chronic spontaneous urticaria in Chinese individuals (Guo et al., 2015). These SNPs are ethnic-specific as they have only been identified in Oriental individuals, but they do not occur in Caucasian individuals according to the 1000 genomes website. Additional clinical associations for *FCER1B* SNPs, all related to the SNP rs569108, include increased risk of developing asthma in Chinese individuals (Ramphul et al., 2014; Hua et al., 2016), and atopic allergy in individuals from Philippines (de Guia et al., 2015). A meta-analysis of 24 studies also supports association of the *FCER1B* rs569108 SNP with asthma, although the risk seems to be restricted to East-Asian individuals (Yang et al., 2014).

Additional clinical associations for *FCER1G* include a weak effect on food sensitization, which is associated with the interactive effect of the *FCER1G* rs2070901 SNP with other SNPs in the *IL4, FCER1B*, and *CYP24A1* genes and cord blood 25(OH) D (Liu et al., 2011).

Strengths of this study include a high number of patients with selective NSAID-hypersensitivity (*n* = 314). Moreover, the clinical phenotypes of these patients, including the proportion of each gender, ages, culprit drugs, and patients' clinical presentations correspond to those described previously among Spaniards, thus indicating that the patient group is representative. The number of patients and controls is sufficiently high to obtain a good statistical power, which is required to obtain conclusive evidence. Limitations in this study include a low number of patients for some subgroups according to the culprit drug (Table 2), the younger age of control individuals as compared to patients, and the low frequency for some of the SNPs analyzed which were, nevertheless, included in the study because of their functional or clinical impact (Table 4).

The results of this study do not support a major association of *FCER1* genotypes in the risk of developing selective NSAID-induced hypersensitivity. Similar genotype distributions and allele frequencies were observed among patients and controls, and the genotypes and frequencies correspond to those previously reported among Spanish patients (Agúndez et al., 2012; Amo et al., 2016). Similarly, no major differences in histamine-metabolizing genes were observed, with the exception of a marginally significant lower frequency of homozygous variant genotypes corresponding to the *DAO* gene variation rs10156191 among patients, which was not significant on comparing allele frequencies (Table 6). The rest of the histamine-metabolism SNPs did not show significant differences when patients and controls were compared, and the genotypes and allele frequencies corresponded with those previously reported for Spanish individuals (García-Martín et al., 2006, 2008, 2015b; Agúndez et al., 2012). Although some gender-related risk associations were identified (summarized in Table 7), in all cases these were due to differences in allele frequencies, but no statistically significant differences for genotypes, in any of the genetic models analyzed, were identified.

In addition to *FCER1A* genes, we analyzed histamine-metabolism genes because these genes, alone or interacting with *FCER1A* genes, may be involved in, and hence modulate, the events that occur downstream of reaction triggering. Again, to our knowledge no previous studies addressing the role of histamine-metabolism in these genes in selective NSAID hypersensitivity have been carried out. We previously reported that the non-synonymous variant on the diamine oxidase gene, rs10156191, which causes decreased metabolic capacity, was significantly associated with cross-intolerance to NSAIDs (OR, 1.7; 95% CI, 1.3–2.1; *Pc* = 0.0003; (Agúndez et al., 2012)). Conversely, in the present study we did not find any association of this SNP with overall selective hypersensitivity. Regarding *HNMT*, it has been reported that the 939A>G polymorphism, which lowers HNMT enzymatic activity by decreasing HNMT mRNA stability, is associated with aspirin intolerant chronic urticaria (Kim et al., 2009). This aside, no other studies analyzing the possible role of gene-related histamine metabolism in NSAID-hypersensitivity have been published. The lack of association of selective NSAID-hypersensitivity with functional histamine-metabolizing SNPs observed in our study is somewhat unexpected because the culprit NSAIDs implicated in both selective and cross hypersensitivity reactions are the same. However, it should be taken into consideration that the mechanisms involved in cross-intolerance are completely different from those involved in selective hypersensitivity (Agúndez et al., 2012) and this may explain the differences in the linkage of histamine-related genes with the clinical entities.

Phenotype-genotype interaction, however, may be relevant to selective NSAID hypersensitivity: We analyzed by linear regression the putative interaction of all genotypes as well as phenotypic factors. The most relevant phenotypic factor was previous history of atopy, which was strongly related to hypersensitivity to ibuprofen. Age was related to hypersensitivity to ibuprofen. Gender was related to the risk of developing hypersensitivity to ASA, with men showing increased risk, which is unexpected because drug allergy is more frequent in women (Doña et al., 2011). This drug-specific phenotypic feature deserves further investigation. This study also revealed the association of the *DAO* rs2052129 GG genotype with hypersensitivity to ibuprofen and a weak association of the *DAO* SNP rs10156191 in homozygosity for the detrimental allele (TT) to an increased risk of developing selective hypersensitivity to ASA, but not to other NSAIDs. The fact that we observed a statistically significant association after multiple regression with only 19 patients with selective hypersensitivity to ASA suggests that the association is strong. Although we should be cautious with regard to this association because of the low number of individuals carrying the TT genotype, further studies are warranted. Additional studies focusing on genes known to be related to organ-specific NSAID-induced hypersensitivity, such as hepatotoxicity (Lucena et al., 2008, 2010; Andrade et al., 2009; Agúndez et al., 2011), should also be conducted in subgroups of selective responders to NSAIDs stratified according to the culprit drug.

## Author contributions

Conceived and designed the experiments: EG Performed the experiments: GA and GE. Analyzed the data: JA and EG. Wrote the paper: EG and JA. Patient assessment: MB, JG, CC, JC, MT, CrM, NB, and GC. Acquisition of data: GA, JG, CC, JC, MT, CaM, NB, GC, GE, MB, JA, and EG. Statistical analysis: AR, JA, and EG. All authors participated in the critical review of the manuscript.

## Funding

This study was financed by grants PI12/00241, PI12/00324, PI15/00303, RETICS RD12/0013/0002, and RETICS RD16/0006/0004 from Fondo de Investigación Sanitaria, Instituto de Salud Carlos III, Spain, and GR15026 from Junta de Extremadura, Spain. Financed in part with FEDER funds from the European Union. The authors are grateful to Professor James McCue for assistance in language editing.

### Conflict of interest statement

The authors declare that the research was conducted in the absence of any commercial or financial relationships that could be construed as a potential conflict of interest.
